# Two Cases of Mucous Papilloma with Carcinomatous Degeneration in the Rectum

**Published:** 1888-03

**Authors:** J. McFadden Gaston

**Affiliations:** Professor of Surgery in the Southern Medical College, Atlanta, Georgia


### TWO CASES OF MUCOUS PAPILLOMA WITH CARCINOMATOUS DEGENERATION IN THE RECTUM.*

---

BY J. McFADDEN GASTON, M. D.,

Professor of Surgery in the Southern Medical College, Atlanta, Georgia.

---

For a better understanding of the details connected with mucous papilloma presented in two cases recently under my care, I would draw attention to a feature of the connection of the rectum with the sigmoid flexure of the colon, which is not brought out with sufficient prominence in our standard works on anatomy

* Read before the Atlanta Society of Medicine, February 7th, 1888.

and physiology. At the upper limit of the rectum there exists a well-defined set of circular fibers in the muscular tissue constituting a sphincter, which ordinarily presents a barrier to the descent of the fœcal matter and gaseous emanations from the large intestine. This may be considered a superior sphincter, and is the division between the semi-fluid contents of the colon and the more solid deposition which is ready for expulsion by the act of defecation. Should this solid mass accumulate above the contractile band, the natural result is impaction of the lower bowel, and it is a fair inference that the tone or capacity of resistance of this structure being impaired, there shall result more or less degeneration of the adjoining papillary tissue. This may ultimately become developed into indurated masses forming tumors.

Upon examining various authorities on rectal diseases, I have not encountered any satisfactory description of the developments which are found in the cases of which I present a report, and there may be grounds for doubt as to their proper classification; my object will have been attained if some colleague should, in view of the facts, be able to clear up the diagnosis.

The almost identical characteristic of the objective phenomena afforded in the two cases renders the description of one, to a great extent, suited to the other, and only requires the addition of some complications in the latter to convey a correct impression of both.

On November 24th, 1887, my attention was called by Dr. G. G. Roy to the case of a gentleman fifty years old, whose general appearance was ruddy, without being plethoric, and indicated average general health. He gave a history of rectal trouble for several months, and had been subjected to carbolic acid injections on several occasions by a specialist in this class of disorders under the impression that he was laboring under hemorrhoids high up in the rectum. But not observing any permanent benefit from this mode of treatment, an examination was desired to determine the nature of the disorder and the prospect of relief. Exploration by the index finger detected an indurated nodular mass around the recto-colic junction, excepting a small space on the right border. The contractility of the sphincter separating the rectum from the colon was destroyed by the deposition of indurated tissue, and the opening was entered with difficulty by the point of the finger, owing to this constriction. Connected with these nodular masses there were ear-like projections extending downward into the rectum, and along the anterior face of this canal there extended downward a prolongation of the induration to a point midway between the constriction above and the anus. The remaining surface of the rectum did not present any marked departure from the normal mucous membrane, and the disease seemed to be confined to the limits above given.

The use of the anal speculum did not indicate any ulceration, though a sanguinolent discharge, mixed with mucus, led us to suspect some ulcerative process higher up in the bowel. As the case was turned over to me for treatment, with a diagnosis of mucous papilloma, having a tendency to carcinomatous degeneration, I determined to give the patient, internally, an alterative course of Donavan's solution, and prescribed five drops three times a day, with a gradual increase until he should reach ten drops thrice daily, and to continue taking this quantity. The local treatment consisted of belladonna ointment one ounce, iodine five grains, and iodide of potash thirty grains, applied upon pledgets of cotton, through the speculum daily in contact with the indurated masses, and kept in position by tamponage with absorbent cotton in the space below. A suppository containing half a grain of opium was placed within the lower part of the rectum to secure against the expulsion of the dressing, and he found relief thus from the tenesmus which had accompanied the disease, so that the pledgets of cotton were usually retained from six to ten or twelve hours. He was more comfortable while the dressing remained than after its discharge, and upon allowing a suspension of the application on Sunday, he requested that it should not be interrupted subsequently, as he could better observe the Sabbath, in its true character of a day of rest, with the constant daily medication.

This course was kept up until December 15, when there was some indication of irritation of the mucous membrane of the small intestines, which was attributed to the full doses of ten drops, three times a day, of Donavan's solution, and it was suspended. For the relief of this disturbance of the bowels, he took the subnitrate of bismuth with mucilage of gum arabic every three hours during several days; and having subsequently a slight difficulty in evacuating his bladder, camphor water, with sweet spirits of nitre, was used as occasion required with benefit.

In view of the softening of the nodules and the diminution of their size up to a certain time, without manifesting any further effect from the continuation of this local measure, I concluded to adopt a more energetic resolvent. Without any guide to a special remedy, I acted upon general principles in my choice. On December 29th, the following prescription was made for local use:

℞. Iodine.

Carbolic Acid.

Tannin, . . . . . . . . . . . . . āāʒi.

Glycerine, . . . . . . . . . . . . f ℥i.

Mix, with heat, for application locally.

This combination was applied upon pledgets of cotton daily to the indurated masses in the upper portion of the rectum, with the use of a speculum, and a tampon of cotton placed below so as to shield the sound mucous membrane of the rectum from the medication.

It was evidently attended with benefit in the reduction of the saliency of the nodules and the diminution of the muco-sanguinolent discharge, and, with only an occasional interruption of a day, was continued until January 17th, 1888. At this time the patient was attacked with influenza, which kept him from coming to my office for five days, when an examination revealed considerable augmentation in the area occupied by the induration, with a reproduction of the ear-like margins and extension downwards along the anterior wall of the rectum. It was then thought best to return to the alterative treatment, and I directed three drops of Donavan's solution to be taken three times a day continuously.

The local application of the compound solution was now made with a camel's hair pencil directly to the surface of the indurations, brushing them over several times at each dressing daily, and cotton was subsequently applied over the parts so as to retain the medication. This course was kept up regularly until February 1st, when some indications of excoriation induced its suspension on alternate days, until on the 6th I became satisfied that no radical change in the nature and extent of the indurated masses was likely to occur from the continuance of this application.

The patient was informed, after a careful examination, of the probable malignant nature of the tumor, and in view of the favorable reports given of the injection of spirits of turpentine in a number of cases diagnosticated as carcinomatous, I advised him to submit to an experiment with this in the mass extending lowest down in the rectum, and if attended with benefit to have it used in the texture of the nodular indurations around the superior sphincter and in the upper portion of the rectum. It was understood that a 4 per cent. solution of cocaine should be injected into the tissue to lessen the sensibility prior to the introduction of the spirits turpentine, as I have observed the marked relief from suffering which it affords in this application.

At 3.30 p. m., February 7, a half drachm of the cocaine solution was injected with a hypodermic needle into the tumor on the anterior face of the rectum, and, waiting a few minutes, ¼ drachm of pure spirits turpentine was injected into the same locality.

The other patient, with a similar development of nodular masses in the upper rectum and encroaching upon the recto-colic opening so as to induce partial stricture, is a gentleman sixty years old, from one of the lower counties of Georgia, who was placed under my care by Dr. J. R. Burton, and presented the appearance of having suffered considerable impairment of his vital forces.

On December 12th, 1887, he was examined by the finger and with the speculum, leading to the conclusion that the developments were of the same nature as those described in the previous case, so that the measures adopted internally and locally were such as detailed in that case, excepting that compound tincture of gentian was combined with the Donavan solution, and occasionally it was requisite to give the fluid extract of cascara sagrada with the subsequent employment of enemata of warm soap-water to secure evacuations from the bowels.

The effects were similar in the temporary relief of the local indurations, but finally there was a limit reached beyond which no further salutary result was secured.

Having suspended the alterative, owing to the irritation of the mucous membrane of the bowels, accompanied with mucous evacuations, the subnitrate of bismuth was resorted to for its relief with a satisfactory result.

*January 1st, 1887.*—An examination over the iliac region revealed an induration along the sigmoid flexure of the colon, and my impression, from the exploration with my finger and the use of a probang, was that this was an extension of the rectal induration. An external application of collodion 1 ounce, tinct. iodine 2 drachms and camphor 1 drachm was made daily over the surfaces corresponding to the tumor in the iliac region until his departure on the 13th of January, 1888.

For the subsequent progress of the case I am indebted to Dr. Burton, who resumed his charge of the patient with the resumption of the internal use of three-drop doses of Donavan's solution, with comp. tinct. of gentian three times a day, and the continuation of the local use of the compound glycerine solution within the rectum. It was stated to the patient, and I wrote to his physician, that there were indications of carcinomatous degeneration, which might call for colotomy if occlusion of the bowel occurred, and thus there might be a prospect of extirpating the diseased structure by a subsequent operation.

After his return home Dr. Burton wrote me an encouraging letter, reporting that after a diarrhœa the induration in the iliac region had almost disappeared, and this led to his inference that it had resulted from fœcal impaction. But he states, in another letter of February 1st, that he found the induration in the sigmoid flexure very apparent and hard, and, extending his examination over the course of the colon, found them very distinct in the transverse portion. He states also that the inguinal glands on the eft side are enlarged and tender.

rectal tube was used to throw up warm water, but without inducing evacuation from the bowels. Copious and repeated enemata of warm water, with additions of castor oil and spirits turpentine, were recommended for the relief of the impaction. All that remains to be done, in my view, is to give an outlet to the contents of the colon by colotomy, as there is sufficient evidence of the arrest of the fœcal matter from the increase of the growth above the recto-colic junction to warrant this operation without further delay.

These cases are presented with a hope that some one may be able to enlighten me as to their further management, and I will report the final result of both at a future day.

*February 16th, 1888.*—A further notice of these cases up to date may be added as the proofs of the report go to the printer.

The patient upon whom the injection of spirits of turpentine into the indurated structure was used on the 7th inst. suffered the most intense pain from the application, notwithstanding the previous injection of cocaine. It was requisite to make hypodermic injections of morphia, gr. $\frac{1}{4}$ and atropia gr. $\frac{1}{150}$ twice, with interval of half an hour before any relief was afforded. He has, however, dispensed since with the use of opiates in any form, though the local inflammatory developments necessitated his remaining in bed continually and led to considerable constitutional disturbance for several days subsequently.

Upon visiting the patient on the 13th inst., there was still sensitiveness upon pressure externally in front of the anus, and the discomfort in the rectum was materially increased by rising up into a sitting posture, while his pulse was ninety beats to the minute and his temperature 101 degrees, Fahrenheit. As these symptoms called for an antifebril treatment he was ordered small doses of tincture of aconite; and his bowels not having responded to the employment of enemata, he was directed to take a teaspoonful of cream of tartar, with half a teaspoonful of sulphur, at night, which secured evacuations.

Since that time there has been less of the local and general disturbance, so that I found him to-day entirely free from fever; and upon a digital examination of the rectum it was evident that there was a favorable change in the tumor extending along its anterior wall, while the nodular induration in the upper posterior portion of the rectal cavity is somewhat more circumscribed than before the application of the spirits of turpentine.

This is the tenth day since the injection into the tumor, and from the gradual subsidence of the inflammation it is expected that in another week he may be ready to submit to a repetition of it in the indurated structure higher up in the rectum.

A letter from Dr. J. R. Burton, of the 11th inst., states that after using the long tube for enemata with the other patient, he has been taking daily doses of the fluid extract cascara sagrada, which caused frequent evacuations, "the actions being always fœcal." On introducing the probang some days previous to his writing, there were coagula discharged—"perhaps an ounce." Since that time the swabs are always stained, but there has been no discharge of blood. The tumor is softer and diminishing in size. The tumors in the tract of the colon have disappeared, except that in the sigmoid flexure, which remains, but is smaller. Upon the whole, he thinks his condition somewhat improved within the last week.

